# Predictors of Early-Recurrence Atrial Fibrillation after Catheter Ablation in Women and Men with Abnormal Body Weight

**DOI:** 10.3390/jcm10122694

**Published:** 2021-06-18

**Authors:** Jan Budzianowski, Jarosław Hiczkiewicz, Katarzyna Łojewska, Edyta Kawka, Rafał Rutkowski, Katarzyna Korybalska

**Affiliations:** 1 Department of Cardiology, Nowa Sól Multidisciplinary Hospital, 67-100 Nowa Sól, Poland; jhiczkiewicz@uz.zgora.pl (J.H.); katarzyna.lojewska@poczta.onet.pl (K.Ł.); 2 Collegium Medicum, University of Zielona Góra, 65-046 Zielona Góra, Poland; 3 Department of Pathophysiology, Poznan University of Medical Sciences, 60-806 Poznań, Poland; ekawka@ump.edu.pl (E.K.); rrutkowski@ump.edu.pl (R.R.); koryb@ump.edu.pl (K.K.)

**Keywords:** catheter ablation, atrial fibrillation, obesity, early recurrence, biomarkers

## Abstract

Our study aimed to select factors that affect the rate of early recurrence (up to 3 months) of atrial fibrillation (AF) (ERAF) following pulmonary veins isolation (PVI) in obese women and men. The study comprised 114 patients: 54 women (age: 63.8 ± 6.3, BMI 31 ± 4 kg/m^2^), and 60 men (age: 60.7 ± 6.7; BMI 31 ± 3 kg/m^2^) with paroxysmal, persistent and long-standing persistent AF. They had been scheduled to undergo cryoballoon (men *n* = 30; women *n* = 30) and radiofrequency (RF) ablation (men *n* = 30; women *n* = 24) using the CARTO-mapping. The blood was collected at baseline and 24 h after ablation. The rate of ERAF was comparable after cryoballoon and RF ablation and constituted 18% in women and 22% in men. Almost 70 parameters were selected to perform univariate and multivariate analysis and to create a multivariate logistic regression (MLR) model of ERAF in the obese men and women. The MLR analysis was performed by forward stepwise logistic regression with three variables. It was only possible to create the MLR model for the group of obese men. It revealed a poor predictive value with an unsatisfactory sensitivity of 31%. Men with ERAF: smokers (OR 39.25, 95% CI 1.050–1467.8, *p* = 0.0021), with a higher ST2 elevation (OR 1.68, 95% CI 1.115–2.536, *p* = 0.0021) who received dihydropyridine calcium channel blockers (OR 0.042, 95% CI 0.002–1.071, *p* = 0.0021) less frequently. Our results indicate a complex pathogenesis of ERAF dependent on the patients’ gender.

## 1. Introduction

It is estimated that ERAF occurs in up to 20–50% of patients after ablation procedures and is considered to be a strong predictor of late recurrence of atrial fibrillation (LRAF) [1,2]. The mechanism of ERAF in patients with obesity is not fully explained in the literature. Nowadays, the origin of this disorder can be explained by: a transient acute inflammation caused by the application of the cryoenergy and radiofrequency (RF) current, a temporary imbalance in the functioning of the autonomic nervous system, a delayed effect of RF current application due to the scar maturation after ablation [1]. Furthermore, the initially incomplete pulmonary vein (PV) isolation is considered as the cause of ERAF [1]. Consequently, obese patients experience electrophysiological and structural atrial changes, the so-called atrial remodeling [1]. In addition, obesity is accompanied by subclinical inflammation and the adipose tissue itself is a source of inflammatory mediators [3]. The epidemiological data suggest a strong correlation between obesity, the impaired left ventricular diastolic function and AF. The increased left atrial (LA) pressure and dimension in obese patients are associated with a longer refraction duration in LA and PVs [1].

Taking into account the increasing number of obese men and women with cardiovascular complications, we decided to select the predictors that affect the rate of ERAF (up to 3 months) following PVs cryoballoon and RF ablation.

## 2. Materials and Methods

### 2.1. Study Population

The study group of 114 patients: 60 men (age 60.7 ± 6.7 years; BMI 30.9 ± 2.7 kg/m^2^) and 54 women (age 63.8 ± 6.3 years; BMI 31.4 ± 4.3 kg/m^2^) with abnormal body weights (BMI > 25 < 40 kg/m^2^; mean BMI 31 ± 3 kg/m^2^, min 29.8, max 38.7, median 32.5), age (>18 and <80; mean age 62 ± 7 years), with documented symptomatic paroxysmal, persistent and long-standing persistent AF, who were scheduled to undergo cryoballoon and RF ablation using the CARTO-mapping at the Cardiology Department in the Multidisciplinary Hospital in Nowa Sól, Poland. The first PVs isolation was performed in 77 patients. The same procedure was performed for the second time in the case of 34 patients and for the third time in the case of 3 patients. Obesity is defined as having a BMI of >25 kg/m^2^ <40 kg/m^2^.

The exclusion criteria in this study were as follows: thrombus located in the LA appendage, acute or chronic infection, diabetes, antibiotic therapy, malignancies, heart failure exacerbation or cardiac surgery, stroke and acute coronary syndromes over the past 3 months.

All the patients studied first underwent a detailed interview with an assessment of arrhythmia symptoms (EHRA scale), comorbidities and current medication. A thorough physical examination was carried out (height, body mass, temperature and blood pressure). The BMI index was calculated as a person’s weight in kilograms divided by their height in metres squared. Waist circumference (WC) was measured midway between the lower rib margin and the iliac crest. Pre-procedural transthoracic and transoesophageal echocardiography (TEE) were performed in all the patients prior to ablation.

**Figure 1:**
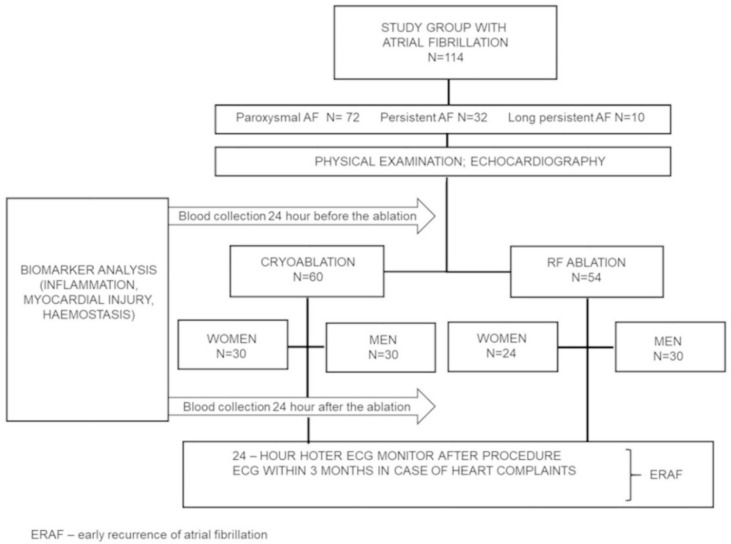
Flowchart of the study.

The study protocol was approved by the Medical Ethics Committee at the Poznań University of Medical Sciences (Approval 44/16) while all the patients signed a written consent for participation. The study was carried out between May 2016 and March 2018. All the participants fulfilled the criteria and completed the study. The study flow chart is presented in Figure 1.

Statistical methods estimate that the total sample size required for the study is 93 patients to ensure the power of the test is 90% at 5% level of significance. The presentation of the subgroups of men and women resulted from the fact that the studied groups were homogenous in terms of the number of patients undergoing RF and cryoablation (60 vs. 54) and comparable representation of men compared to women both undergoing only cryoablation (30 vs. 30) and only RF ablation (30 vs. 24). There was a lower number of women undergoing RF ablation (24). Initially, we planned 30 vs. 30, but some women did not qualify for the study.

### 2.2. Radiofrequency Ablation

RF ablation was performed point-by-point in accordance with the guidelines [1]. PV isolation was performed using the focal ablation strategy guided by the CARTO 3-D mapping system (Biosense Webster, Diamond Bar, CA, USA). All the procedures were performed under conscious sedation with local anasthetic. The double transseptal puncture was performed following the fluoroscopic guidelines. Immediately after the puncture, intravenous unfractioned heparin (UFH) was administered to maintain the target activated clotting time of 300–350 s. PV isolation was performed using 7F Navistar ThermoCool and 8F ThermoCool SmartTouch SF (Biosense Webster). In five patients RF ablation was performed using the “ablation index” algorithm.

### 2.3. Cryoballoon Ablation

Cryoablation was performed as previously reported [2]. The second generation 28 mm cryoballoon (Arctic Front Advance, Medtronic, Minneapolis, MN, USA) was used. The venous delivery of the cryoballoon was managed using a 15F steerable sheath (FlexCath Advance, Medtronic). The correct position of the cryoballoon was confirmed by contrast retention in the PVs. The cryoapplication process lasted 180–240 s per vein and was verified by the circular mapping catheter (CMC, Achieve™; Medtronic, Minneapolis, MN, USA) to confirm electrical isolation. During the application in the right veins, the diaphragmatic nerve was constantly stimulated (30/min) to avoid its paralysis.

### 2.4. Biochemical Analyses

The venous blood was collected at baseline and 24 h after ablation. All routine biochemical analyses (D-dimer, fibrinogen, INR, aPTT, hsTnT, CK, CKMB, CRP) were performed immediately in a central hospital laboratory. In the analysis the peripheral blood count was marked with CELL-DYN Ruby (Abbott Diagnostics, Santa Clara CA, USA USA). D-dimer, fibrinogen, INR and aPTT were tested using STACompact Max (Diagnostica Stago, Parsippany, NJ, USA). High sensitivity TnT (hsTnT) was marked with a Cobas c601 device (Roche Diagnostics GmbH, Mannheim, Germany). CK was analyzed using a kinetic serum test while CKMB was analyzed with CKMB immunoassay concentrations. CRP was inspected with an immunoturbimetric latex CRP assay (Roche Diagnostics GmbH Mannheim, Germany).

The additional samples of serum were aliquoted and stored at −80 °C until assayed. The following parameters were measured using DuoSet Immunoassay Development Kits (R&D Systems, Minneapolis, MN, USA) according to the manufacturer’s instructions: hsIL-6, pentraxin (PTX), von Willebrand factor (vWF), thrombomodulin (TM), sICAM-1, sVCAM-1, t-PA, PAI-1, ST2, leptin, adiponectin. The sensitivities of the assays are presented in Table S1.

### 2.5. Post-Ablation Management and Follow-Up

The patients were monitored for the first 24 h after ablation using a 24-h Holter monitoring in an outpatient clinic to evaluate ERAF within 3 months after ablation (Mortara Instrument, Milwaukee, WI, USA). Additionally, a 12-lead electrocardiogram (ECG) was recommended for the patients with symptoms of arrhythmia. Antiarrhythmic drugs (AAD) were not routinely used after ablation except for the highly symptomatic patients with ERAF. Oral anticoagulants were continued for at least 2 months. The decision to continue anticoagulation was based on an individual’s stroke risk determined by the CHA_2_DS_2_-VASc score.

### 2.6. Echocardiogram

Transthoracic echocardiography (TTE) (iE33, Philips Medical Systems, Andover, MA, USA), was performed with a two-dimensional Doppler assessment in typical projections in accordance with the American Echocardiographic Society and the European Association of Cardiovascular Imaging [4]. The left ventricle ejection fraction was assessed by a modified Simpson’s rule. The LA volume index was calculated using the disk summation technique.

### 2.7. Statistical Analysis

Statistical analysis was performed using Statistica 12.0 software (StatSoft, Tulsa, OK, USA) and GraphPad PrismTM 6.00 (GraphPad Software Inc., La Jolla, CA, USA). The student’s *t*-test was used to test the significance of the assessed parameters before and after the procedure. For variables not normally distributed, a Wilcoxon signed rank test was used to compare the patients within a group and a Mann-Whitney test was used to compare the groups with each other. The normal distribution of continuous variables was tested with the Shapiro–Wilk and the D’Agostino & Pearson tests. Pearson and Spearman correlation analysis was used for assessing the correlation depending on the data distribution. Contingency was analyzed with a Chi-square test.

Multivariate logistic regression (MLR) analysis was performed to identify the logistic regression model of ERAF predictors. The MLR model was built using the forward stepwise logistic regression method. Only a limited number of variables (out of 66 available variables) was used to create the MLR model in accordance with statistical rules. The variables that could potentially be associated with the occurrence of ERAF were used based on the literature and clinical experience. A *p*-value < 0.05 was considered significant. Continuous parameters were expressed as means standard deviation and categorical variables as numbers and percentages.

## 3. Results

### 3.1. Patient Characteristics

A total of 114 patients with abnormal body weight and symptomatic, refractory AF treated with cryoablation (60 patients; 30 women, 30 men) and RF ablation (54 patients; 24 women, 30 men) participated in the study. The baseline characteristics of all the women and men have been summarised in Table 1. The study flow chart is presented in Figure 1. As demonstrated in Table 1 persistent AF was significantly more frequent in the group of men (19% vs. 37%). Other parameters relevant to the procedure were comparable in both groups (EHRA score, HAS-BLED, LA Volume, LAVI, EF), except for the CHA_2_DS_2_-VASC score.

The patients were matched according to their age and BMI, but the men tended to have a higher body weight, WC, and a five times lower leptin concentration than the women. Dyslipidemia was more pronounced in the female group which is a consequence of the (i) abnormal body weight, (ii) higher incidence of hypothyroidism (significant increase in TSH in women) and (iii) menopause. The male group was characterized by higher morbidity due to coronary artery disease (CAD), which resulted in higher ST2, hs-TnT and vWF levels. The group of women was slightly older than the group of men, which probably resulted in their lowered GFR.

Physiologically females have a higher level of fibrinogen than males, which among many other factors, predisposes them to a higher risk of thromboembolism and a higher CHA_2_DS_2_-VASC score. The women were more frequently treated with vitamin K antagonists monitored by INR, which was higher in their group. The men used non-vitamin K antagonist oral anticoagulants more often than the women.

### 3.2. Ablation Procedure—Early and Late Recurrence of AF

As opposed to cryoablation, RF ablation was associated with a higher number of applications, significantly longer procedural and application time but shorter fluoroscopy duration (Table 2). ERAF occurred in 20% of all the treated patients (23 with ERAF out of 114). There were no differences between women and men in the rates of ERAF (18% vs. 22%) (Table 3). The percentage of ERAF was similar after cryoballoon and RF ablation (Table 2). Furthermore, the rate of paroxysmal ERAF was similar to the rate of persistent ERAF after both procedures (Table 2). The females treated with RF ablation were older than men (64 vs. 58 years) and had a higher BMI when treated with cryoablation (32.8 vs. 30.5; Table 2).

**Figure 2:**
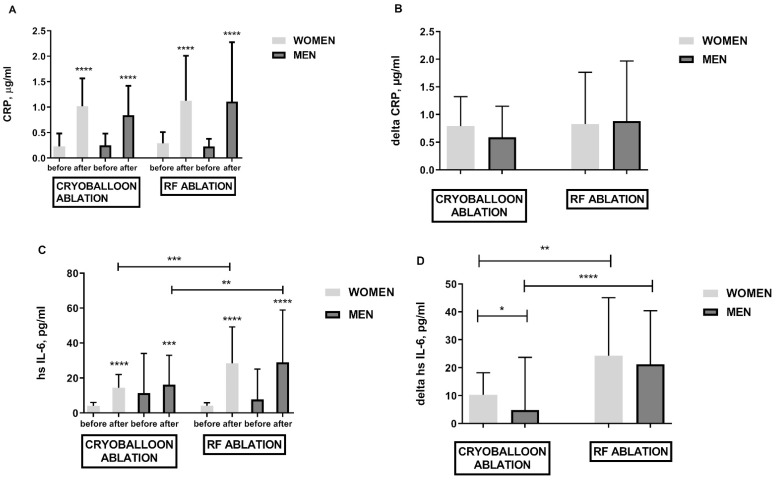
Biomarkers of inflammation in obese females and obese males following cryoballoon and RF ablation; panel (**A**)—CRP elevation in response to cryo and RF ablation; panel (**B**)—CRP difference before and after cryoballoon and RF ablation (delta); panel (**C**)—hs Il-6 elevation in response to cryoballoon and RF ablation; panel (**D**)—hs Il-6 difference before and after cryoballoon and RF ablation (delta). Asterisks represent a significant difference: * *p* < 0.05, ** *p* < 0.01, *** *p* < 0.001, **** *p* < 0.0001.

Both methods of treatment triggered inflammation which was confirmed by the increased values of CRP inflammation markers (Figure 2A,B), hsIL-6 (Figure 2C,D), WBC (data not shown), and pentraxin (data not shown). There was no difference between the elevation of inflammatory parameters such as CRP in both procedures. Only the evaluation of high sensitivity parameters such as hsIL-6 did show that RF ablation generates more intense inflammation than cryoballoon ablation (Figure 2C,D). It was shown that the women treated with cryoballoon ablation developed greater inflammation resulting in higher hsIL-6 delta after the procedure (Figure 2D).

**Figure 3:**
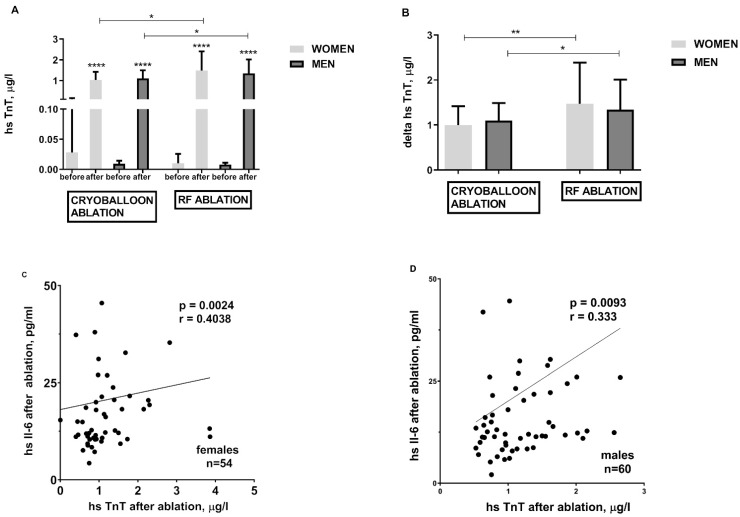
Troponin concentration before and after cryoballoon and RF ablation and its relationship with ablation-induced inflammatory process in obese females and obese males; panel (**A**)—hs TnT elevation in response to CB and RF ablation; panel (**B**)—hs TnT difference before and after cryoballoon and RF ablation (delta); The relationship between the degree of troponin release and the intensity of inflammation in the group of females panel (**C**) and males panel (**D**). Asterisks represent a significant difference: * *p* < 0.05, ** *p* < 0.01, **** *p* < 0.0001.

Both cryoballoon and RF ablations are inherently associated with cardiomyocyte damage and release of hs-TnT (Figure 3A), CPK (data not shown), and CK-MB (data not shown). However, RF ablation engenders greater cardiomyocyte damage (Figure 3A,B). When it comes to atrial myocardial injury and hs-TnT release, both are proportional to the hsIL-6 concentration regardless of gender (Figure 3C,D).

Out of 114 patients, 32 (28%) of them also experienced AF late recurrence (LRAF) assessed by a 24-h Holter monitoring at 6-, 9-, and 12-month intervals following ablation (authors’ observation). The number of patients with ERAF was comparable to the number of patients with LRAF (ERAF 20% vs. LRAF 28%, *p* = 0.1636). Among 23 patients with ERAF, 16 of them (70%) also had LRAF (70% vs. 30%, *p* = 0.008) (data not shown).

### 3.3. Predictors of ERA—Univariate Model

Since there was no difference in ERAF and its occurrence in both methods (Table 2) and in both groups of obese women and men (Table 2 and Table 3), we decided to divide all the females and males into two groups (with and without ERAF) and compare them (Table 3), forming an univariate model. Among many analyzed parameters in both men and women with ERAF, only two parameters distinguished them from those without ERAF. In the group of men and women, it was always a higher response to the ablation procedure (delta). Also, a higher value of sICAM-1 characterized the group of women with ERAF while a higher value of ST-2 protein characterized the group of men (Table 3). Both procedures increased the ST2 concentration, but we also documented that higher ST2 levels were particularly characteristic of the men qualified for cryoablation. RF ablation was responsible for a higher ST2 protein production only in the women (data not shown). The obese women had higher sICAM–1 before the treatment than the obese men (data not shown). Therefore, sICAM-1 which characterizes inflammatory activation of endothelium in obesity, could be a determinate parameter for ERAF only in the group of women.

### 3.4. Predictors of ERAF—Multivariate Model

Out of 66 available variables (presented in Table S2) only a limited number of them was used in the MLR analysis, which was performed in several variants for the entire group of obese patients (*n* = 114) and separately for the group of obese women (*n* = 54) and obese men (*n* = 60). We only managed to create three MLR models in the group of men. The model with the best results is presented in Table S3. The results demonstrated that the extent of ST2 protein elevation (OR 1.68, 95% CI, 1.115–2.536, *p* = 0.011), less frequent use of calcium channel blockers (CCB) (OR 0.042, 95% CI 0.002–1.071, *p* < 0.05) and smoking (OR 39.25, 95%CI, 1.050–1467.8, *p* = 0.042) were the independent predictors of ERAF in males. The proposed model of ERAF had a high specificity of 95.74%, and low sensitivity at 30.77%. Summing up, the MLR model is suitable for classifying obese men without ERAF (high specificity) while it is not satisfactory for detecting obese men with ERAF (low sensitivity).

## 4. Discussion

Due to the increasing number of obese patients suffering from an increased recurrence of atrial arrhythmias, we decided to recruit two groups of obese men and women who were qualified for cyoballoon and RF ablation and assess the treatments’ effectiveness based on ERAF. In the examined group of 114 obese patients with matched age and BMI, ERAF occurred in 20% of them with the same frequency in both women and men following both types of ablation. Having two groups of patients, women and men, collecting 66 variables describing the patients’ clinical condition required laboratory tests and ablation procedures. They enabled us to perform three MLR models to find the factor/factors conducive to ERAF three months after ablation. A separate analysis was done for the entire group of obese patients (*n* = 114), the group of obese women (*n* = 54), and obese men (*n* = 60). We only managed to create three MLR models in the group of obese men. As stated above, the results demonstrated that the extent of ST2 protein elevation, less frequent use of CCB and smoking were the independent predictors of ERAF in obese males. The proposed model of ERAF had a high specificity of 95.74%, which indicates that people without ERAF are correctly classified as patients with successful ablation (over 90%). Unfortunately, the model has a low sensitivity of 30.77%, which means that not all obese men with ERAF are detected.

The ST2 protein is associated with inflammation, fibrosis and myocardial overload. We noted a slightly higher concentration of ST2 protein before the procedure in men (probably due to higher CAD morbidity in this group) and its prominent elevation after both ablation methods. The higher ST2 concentration was particularly characteristic of the men qualified for cryoablation. The large population in the Framingham Heart Study also demonstrated higher ST2 levels in men [5]. The above trend may be related to sex hormones. It has been proven that in the group of postmenopausal women, without cardiovascular diseases, the concentration of ST2 was significantly lower when compared to the men of similar age [6]. In the study of Okar et al. [7], in MLR, the ST2 protein was an independent predictor of AF recurrence after cryoablation due to paroxysmal AF [7].

Smoking is a widely recognized factor leading to more than a two-fold increased risk of AF [8]. Smoking increases the incidence of nonPV triggers in patients with persistent AF. Smokers who had arrhythmia triggers located in the right atrium had a worse outcome after ablation [9].

The last MLR model seemed to be the most unexpected. It is linked to the less frequent use of CCBs dihydropyridine antihypertensive drugs in males with ERAF. Given the characteristics of the study group—elderly, obese patients with hypertension (71%), CAD (12%), and dyslipidemia (35%), this type of medication is commonly used. However, it is worth emphasizing that dihydropyridine CCBs lack an antiarrhythmic effect [10].

The results of ablation in women and men are inconclusive. Previous studies revealed that gender affects the recurrence rate of AF after catheter ablation. In large multicenter studies, female patients had a lower long-term efficacy than males [11,12]. However, Andrade et al., in the STOP AF trial, reported that the only significant factor associated with ERAF was the male sex [13]. Our MLR analysis performed on all 114 participants reveals that in obese patients, there is no relationship between gender and the recurrence rate of AF after catheter ablation.

Our study confirmed that the obese women had an 80% higher leptin concentration than the obese men. A higher leptin concentration in women results from a higher percentage of adipose tissue and greater secreted leptin per unit mass of adipose tissue. Leptin directly modulates atrial myocytes’ electrophysiological basis by regulating calcium homeostasis in atrial myocytes, affecting atrial fibrosis and angiotensin II-induced AF [14]. Additionally, we observed a higher body weight and WC in the group of men, resulting from a different distribution of body fat in both sexes. Women had higher incidence of dyslipidemia. Demerath et al., showed a relationship between sICAM-1, sVCAM-1 and the concentration of total cholesterol and HDL cholesterol. This effect may be an explanation for the observed effect of higher concentration of these molecules in women due to observed dyslipidemia and physiologically higher concentration of HDL in women compared to men (Table 1) [15]. Men had higher concentrations of vWF. There is a relationship between vWF and the amount of visceral fat that produces adipokines responsible for endothelial dysfunction [16]. Moreover, the group of men suffered from CAD more frequently than the women (18% vs. 6%), which points at equally frequent atherosclerosis dominated by endothelial dysfunction.

Obesity is associated with a higher recurrence and greater impact of AF. The ESC-EHRA Atrial Fibrillation Ablation Long-Term Registry shows that BMI > 30 kg/m^2^ can increase the recurrence rate of AF after ablation [17]. Nevertheless, no study so far has attempted to evaluate the parameters responsible for ERAF in overweight and obese patients.

The total procedural time, the number and duration of applications were longer in RF ablation than in cryoablation. RF ablation also generated more inflammation, which indicates the significant complexity of this method, the need to perform a precise map of LA and PVs using the CARTO electroanatomical system. However, CARTO practically limits the use of fluoroscopy.

Many authors emphasize that the frequency of ERAF is similar in RF and cryoballoon ablation [18,19], which is also confirmed by our research. The most common parameters that contribute to the recurrence of arrhythmia after ablation are: age [20], LA dimension [18,20], inflammatory markers [18], and reduced troponin levels [2,20]. The studies mentioned above indicate that LA inflammation and an increased LA dimension play an essential role in AF recurrence after ablation. In our study the differences in baseline characteristics could have an impact on clinical outcomes. First, the enrolled women were older and their GFR was lower than the men. Age-related fibrosis and lower GFR are common factors associated with AF recurrence after catheter ablation [20]. Second, a higher incidence of persistent AF in the male group is indeed associated with poor clinical outcomes [1].

Each ablation procedure causes damage to cardiomyocytes, more prominent in RF ablation, which is reflected in higher hs-TnT levels [2,20]. As we presented, the hs-TnT concentration increases proportionally to the inflammatory process both in men and women. We also showed a significantly higher concentration of hs-TnT before catheter ablation in the obese men, which may be associated with a higher incidence of CAD in this group [21].

In summary, we indicated the importance of structural remodeling by demonstrating the increasing ST2 protein concentrations associated with ERAF in obese men. Additionally, the CCB therapy and smoking also seemed to be the important factors contributing to ERAF in this group of patients. It was only possible to create MLR model in the group of the obese men due to the lack of statistical significance in the other two remaining groups (the obese women and the entire group of patients). The MLR model is suitable for classifying obese men without ERAF (high specificity) while not satisfactory for detecting obese men with ERAF (low sensitivity). 

The poor predictive value of the MLR model may indicate the multifactorial nature of ERAF and the limited predictive value of biomarkers. The assessment of inflammation using highly sensitive markers such as sICAM-1, which characterizes the inflammation of the endothelium in obesity, may aid detecting patients more susceptible to ERAF. What is more, the evaluation of myocardial overload and fibrosis, such as the ST-2 protein, may help to select patients with more severe left atrial fibrosis who will potentially be at a higher risk of ERAF. Nevertheless, further research is required when it comes to the assessment of ERAF with more accurate methods of heart rhythm monitoring such as long- term rhythm monitoring and AF burden evaluation, which better reflect the outcomes that are clinically relevant [22].

### Limitations of the Study

Our study was single-centred with a relatively small number of patients. The study group was heterogeneous in terms of the number of ablation procedures and RF ablation technique. ERAF was detected based on clinical symptoms, 12-lead ECG and 24-h Holter monitoring. Therefore, asymptomatic ERAF might have been missed. UFH could have modified the concentration of assessed biomarkers. Due to complex pharmacokinetics, UFH is theoretically absent in the blood collected 24 h after ablation. However, its earlier effect may be detected and it may change the concentration of some of the assessed parameters. Furthermore, the heparinized saline solution (0.9% NaCl) used during the procedure may have had an impact on the dilution of some biomarkers tested after ablation and thus also on their concentration 24 h after the procedure. The decision to collect blood 24 h after procedure was based on the expertise of the researchers who stated that during this time the severity of inflammation and myocardial injury after ablation is the highest [23]. Also, a specific limitation in the interpretation of myocardial injury biomarkers such as CPK and CK-MB occurred due to their thermal instability during RF ablation [21].

## 5. Conclusions

It was only possible to create the MLR model in the group of obese men, but not in the group of obese women. It revealed a poor predictive value with an unsatisfactory sensitivity of 31%, which indicates a poor classification of patients with ERAF following catheter ablation. The males with ERAF, who were smokers, had a higher level of ST2 cardiac stress biomarker in response to ablation. CCBs were less frequently administered in this group. The results demonstrate the multifactorial character of ERAF, which is determined by the gender of the obese patients.

## Figures and Tables

**Table 1:** Baseline characteristics of the studied patients.

Parameter	Females (*n* = 54)	Males (*n* = 60)	*p* Value
Age (years)	63.8 ± 6.3	60.7 ± 6.7	*p* = 0.0660
BMI (kg/m^2^)	31.4 ± 4.3	30.9 ± 2.7	*p* = 0.3827
WC (cm)	97.9 ± 12.8	104.8 ± 10.8	*p* = 0.0017
Leptin, ng/mL	30.7 ± 20.6	6.2 ± 6.0	*p* < 0.0001
CRP, μg/mL	0.25 ± 0.24	0.24 ± 0.19	*p* = 0.7764
INR	1.83 ± 0.77	1.53 ± 0.66	*p* = 0.0253
Fibrinogen, mg/dL	399 ± 86	367 ± 74	*p* = 0.0308
PLT, 10^3^/mL	235 ± 67	197 ± 48	*p* = 0.0008
Haemoglobin, g/dL	14.1 ± 1.1	15.5 ± 1.4	*p* < 0.0001
Glucose, mg/dL	100.8 ± 9.9	102.3 ± 11.1	*p* = 0.3615
Cholesterol, mg/dL	197.0 ± 37.3	173.9 ± 38.4	*p* = 0.0005
LDL, mg/dL	124.7 ± 35.6	111.2 ± 33.9	*p* = 0.0406
HDL, mg/dL	65.5 ± 14.15	54.0 ± 9.8	*p* < 0.0001
GFR, mL/min	67.9 ± 14.7	80.5 ± 14.9	*p* < 0.0001
TSH μU/mL	2.6 ± 2.4	1.6 ± 1.6	*p* = 0.0081
hs-TnT, ng/L	9.1 ± 10.0	9.3 ± 5.0	*p* = 0.0422
vWF, ng/mL	1.81 ± 0.66	2.24 ± 1.01	*p* = 0.0083
ST2, ng/mL	1.5 ± 1.4	1.8 ± 1.9	*p* = 0.0314
Paroxysmal AF, no, (%)	39 (72)	33 (55)	*p* = 0.0854
Persistent AF, no, (%)	10 (19)	22 (37)	*p* = 0.0313
Long-standing persistent AF, no, (%)	5 (9)	5 (8)	*p* = 0.8615
EHRA 1, *n* (%)	0 (0)	2 (3)	*p* = 0.1759
EHRA 2a, *n* (%)	8 (15)	18 (30)	*p* = 0.0537
EHRA 2b, *n* (%)	24 (44)	19 (32)	*p* = 0.1599
EHRA 3, *n* (%)	20 (37)	20 (33)	*p* = 0.6791
EHRA 4, *n* (%)	2 (4)	1 (2)	*p* = 0.4975
Left atrial volume	93.9 ± 25.2	96.4 ± 34.4	*p* = 0.6258
LAVI, mL/m^2^	47.8 ± 11.8	44.1 ± 14.5	*p* = 0.1464
LVEF, %	58.1 ± 3.1	56.7 ± 6.9	*p* = 0.7078
SBP	129 ± 14	126 ± 11	*p* = 0.1997
DBP	77 ± 10	81 ± 11	*p* = 0.0619
Mean CHA2DS2-VASC score	2.4 ± 1	1.5 ± 0.89	*p* < 0.0001
Mean HAS-BLED score	1.3 ± 0.80	1.1 ± 0.68	*p* = 0.0957
**Comorbidities and Medications**			
Hypertension, no, (%)	39 (72)	42 (70)	*p* = 0.7939
Coronary artery disease, no, (%)	3 (6)	11 (18)	*p* = 0.0379
Dyslipidemia, no, (%)	25 (46)	15 (25)	*p* = 0.0174
Heart Failure, no, (%)	1 (2)	4 (7)	*p* = 0.1431
Thyroid disease, no, (%)	19 (35)	11 (18)	*p* = 0.0966
Beta-blocker, no, (%)	47 (87)	47 (78)	*p* = 0.2225
CCB, no, (%)	11 (20)	13 (22)	*p* = 0.8654
NOAC, no, (%)	33 (61)	47 (78)	*p* = 0.0448
VKA, no, (%)	21 (39)	13 (22)	*p* = 0.0264
Statins, no, (%)	23 (43)	25 (42)	*p* = 0.9204
Diuretics, no, (%)	16 (30)	19 (32)	*p* = 0.8139
ACE inhibitor, no, (%)	18 (33)	18 (30)	*p* = 0.7022
ARB, no, (%)	15 (28)	19 (32)	*p* = 0.6504
Anti-arrhythmic drugs, no, (%)	30 (56)	28 (47)	*p* = 0.3432

Continuous data are presented as means ± SD. Categorical data are presented as counts with their percentage values in brackets. BMI, body mass index; W, waist circumference; CRP, C-reactive protein; INR, international normalized ratio; PLT, platelets; GFR, glomerular filtration rate; hs-TnT, high-sensitive cardiac troponin T; vWF, von Willebrandt factor; AF, atrial fibrillation; EHRA, European Heart Rhythm Association; LAVI, left atrial volume index; LVEF, Left ventricle ejection fraction; SBP, Systolic blood pressure; DBP, Diastolic blood pressure; CHA2DS2-VASc, Congestive heart failure, Hypertension, Age ≥ 75, Diabetes, Stroke, Vascular disease, Age 65–74, Sex (female); HAS-BLED, Hypertension, Abnormal renal/liver function, Stroke, Bleeding history or predisposition, Labile INR, Elderly (>65 years), Drugs/alcohol concomitantly; CCB, calcium channel blockers; NOAC, non-vitamin K antagonist oral anticoagulants; VKA, vitamin K anagonist; ACE-I, angiotensin converting enzyme inhibitor; ARB, angiotensin II receptor blocker.

**Table 2:** Procedural characteristics and the type of ERAF after catheter ablation in obese females and obese males.

	Females (*n* = 54)		Males (*n* = 60)	
	Cryoablation (*n* = 30)	RF Ablation (*n* = 24)	Cryoablation (*n* = 30)	RF Ablation (*n* = 30)
Age, years	63 ± 5.8	## 64 ± 6.9	62 ± 5.5	58 ± 9.0 *
BMI, kg/m^2^	# 32.8 ± 3.5	29.8 ± 4.6 **	31.0 ± 2.1	30.5 ± 3.0
Total procedure time, min	105.2 ± 30.2	196.9 ± 52.1 ****	98.6 ± 25.0	199.7 ± 37.7 ****
Fluoroscopy time, min	15.5 ± 5.9	8.3 ± 3.3 ****	14.3 ± 5.4	9.2 ± 4.5 ***
Application time, min	30.5 ± 8.7	54.9 ± 16.2 ****	28.2 ± 8.2	58.4 ± 17.5 ****
Application number	8.2 ± 2.4	47.2 ± 50.9 ****	7.8 ± 2.3	25.2 ± 10.1 ****
ERAF, *n* (%)	5 (17)	5 (21)	5 (17)	8 (27)
Paroxysmal ERAF, *n* (%)	4 (13)	3 (12.5)	3 (10)	4 (13)
Persistent ERAF, *n* (%)	1 (3)	2 (8)	2 (7)	4 (13)

* Significant difference CB vs. RF ablation, # significance difference females vs. males. * *p* < 0.05, ** *p* < 0.01, *** *p* < 0.001, **** *p* < 0.0001; # *p* < 0.05, ## *p* < 0.05.

**Table 3:** Comparison of clinical and laboratory characteristics in obese females and obese males with and without ERAF following cryoballoon and RF ablation.

	Females		Males	
Parameter	(+) ERAF	(−) ERAF	(+) ERAF	(−) ERAF
*n* (%)	10 (18)	44 (82)	13 (22)	47 (78)
Age (years)	64.3 ± 7.7	63.7 ± 6.1	59.1 ± 9.9	60.5 ± 7.1
Smoking, *n* (%)	0 (0)	2 (2)	2 (15)	1 (2)
BMI, kg/m^2^	30.4 ± 3.7	31.7 ± 4.4	31.3 ± 3.5	30.6 ± 2.3
WC, cm	96.1 ± 7.5	98.7 ± 13.3	107.3 ± 12.1	104.4 ± 10.0
leptin, ng/mL	29.9 ± 17.9	30.9 ± 21.4	6.8 ± 4.3	6.1 ± 6.4
Ablation Procedure				
ERAF *n*, (%)	10 (18)	0 (0)	13 (22)	0 (0)
Procedure time, min	139.0 ± 64.4	147.5 ± 61.6	155.3 ± 59.8	147.4 ± 60.6
Cryoablation time, min	97.0 ± 32.1	106.8 ± 30.2	95.8 ± 21.1	99.2 ± 26.0
RF ablation time, min	181.0 ± 62.5	201.1 ± 50.1	192.5 ± 42.0	202.3 ± 36.7
Fluoroscopic time, min	11.4 ± 5.7	12.5 ± 6.2	11.3 ± 5.4	11.9 ± 5.6
Application time, min	35.1 ± 10.8	42.0 ± 18.2	45.2 ± 20.9	42.5 ± 20.4
Number of applications	23.0 ± 33.3	25.1 ± 39.5	17.1 ± 13.7	16.2 ± 10.9
Cryoablation, *n* (%)	5 (50)	25 (57)	5 (38)	25 (53)
RF ablation, *n* (%)	5 (50)	19 (43)	8 (62)	22 (47)
Cardiovascular Parameters				
LA volume, mL	91.8 ± 27.2	94.3 ± 25.1	106.8 ± 30	93.9 ± 35.1
LAVI, mL/m^2^	46.7 ± 11.4	48.7 ± 12.0	49.9 ± 15.0	42.8 ± 14.6
EF, %	58.1 ± 2.8	58.1 ± 3.2	57.2 ± 9.7	56.6 ± 6.2
CHA2DS2-VASC score, mean ± SD	2.4 ± 1.3	2.4 ± 1.0	1.6 ± 1.0	1.5 ± 0.9
HAS-BLED score, mean ± SD	1.3 ± 1.1	1.3 ± 0.7	1.1 ± 0.8	1.1 ± 06
SBP, mmHg	130 ± 14.6	129 ± 13.6	125 ± 11.9	127 ± 11.2
DBP, mmHg	78 ± 12.4	77 ± 9.3	83 ± 15.0	80 ± 9.5
Comorbidities and Medications				
Hypertension, *n* (%)	7 (70)	32 (59)	9 (69)	33 (70)
CAD, *n* (%)	1 (10)	2 (4)	1 (8)	10 (21)
Dyslipidemia, *n* (%)	4 (40)	21 (48)	2 (15)	13 (28)
Heart Failure, *n* (%)	0 (0)	1 (2)	1 (8)	10 (21)
Beta Blocker, *n* (%)	9 (90)	38 (86)	11(85)	36 (77)
CCB, *n* (%)	0 (0)	11 (25)	1 (8)	12 (26)
NOAC, *n* (%)	7 (70)	26 (59)	12 (92)	35 (74)
VKA, *n* (%)	3 (30)	18 (41)	1 (8)	12 (26)
Statins, *n* (%)	5 (50)	18 (41)	5 (38)	20 (43)
Diuretics, *n* (%)	3 (30)	13 (29)	3 (23)	16 (34)
ACEI, *n* (%)	3 (30)	15 (34)	4 (31)	14 (30)
ARBs, *n* (%)	4 (40)	11 (25)	2 (15)	17 (36)
AAD, *n* (%)	5 (50)	25 (57)	6 (46)	23 (49)
Laboratory Findings				
Glukose, mg/dL	104.3 ± 8.2	100.0 ± 10.2	105.5 ± 12.7	101.4 ± 10.6
Cholesterol, mg/dL	187.1 ± 27.9	199.3 ± 39.1	165.9 ± 32.2	176.1 ± 39.9
LDL, mg/dL	118.5 ± 27.4	126.1 ± 37.3	104.8 ± 27.1	113.0 ± 35.6
eGFR, mL/min	72.3 ± 18.5	66.9 ± 13.7	81.2 ± 23.4	80.3 ± 11.9
Response to Ablation				
Parameter	delta	Delta	delta	delta
CRP, μg/mL	0.7 ± 0.5	0.8 ± 0.8	1.2 ± 1.6	0.6 ± 0.5
hsIL-6, pg/mL	20.0 ± 19.9	15.7 ± 15.8	14.6 ± 34.5	12.4 ± 14.3
PLT, 10^3^/mL	−40.7 ± 36.6	−45.3 ± 30.5	−29.9 ± 29.8	−29.8 ± 18.7
Fibrinogen, mg/dL	−34.4 ± 47.9	−42.2 ± 87.2	19.7 ± 58.6	−14.7 ± 67.3
D-Dimer, mg/dL	0.5 ± 1.2	0.2 ± 0.6	0.07 ± 0.1	0.1 ± 0.3
Hs-TnT, ng/L	1.1 ± 0.5	1.3 ± 0.8	1.2 ± 0.6	1.2 ± 0.5
CPK, U/L	100.6 ± 85.3	139.1 ± 117.9	35.7 ± 96.4	111.2 ± 130.6
CK-MB, U/L	10.3 ± 10.2	15.9 ± 16.8	9.0 ± 10.8	16.2 ± 18.5
vWF, ng/mL	0.37 ± 0.65	0.11 ± 0.58	0.2 ± 0.5	0.06 ± 0.7
sICAM-1, ng/mL	0.8 ± 14.1 *	−22.2 ± 52.6 *	13.4 ± 73.7	0.03 ± 20.6
ST-2, ng/mL	1.9 ± 4.2	1.5 ± 2.4	2.5 ± 2.6 *	1.1 ± 1.9

Abbreviations: BMI, body mass index; WC, waist circumference; ERAF, early recurrence atrial fibrillation; RF, radiofrequency; LAVI, left atrial volume index; EF, ejection fraction; CHA_2_DS_2_-VASc, Congestive heart failure, Hypertension, Age ≥ 75 (doubled), Diabetes, Stroke (doubled), Vascular disease, Age 65–74, Sex (female); HAS-BLED, Hypertension, Abnormal renal/liver function, Stroke, Bleeding history or predisposition, Labile INR, Elderly (>65 years), Drugs/alcohol concomitantly; SBP, Systolic blood pressure; DBP, Diastolic blood pressure; CAD, coronary artery disease; CCB, calcium channel blockers; NOAC, non-vitamin K antagonist oral anticoagulant; VKA, vitamin K antagonist; ACE-I, angiotensin converting enzyme inhibitor; ARB, angiotensin II receptor blocker, AAD, anti-arrhythmic drugs; GFR, glomerular filtration rate; CRP, C-reactive protein; PLT, platelets; hs-TnT, high-sensitive cardiac troponin T; vWF, von Willebrandt factor; s-ICAM, intercellular adhesion molecul; Delta denotes the response to the ablation procedure. Delta was defined as the change in the biomarker concentration between two assays performed within 24-hour period (after ablation—before ablation). Significance difference ERAF(+) vs. ERAF (−) * *p* < 0.05.

## Data Availability

The data presented in this study are available in this article and supplementary material.

## Supplementary Materials

The following are available online at https://www.mdpi.com/article/10.3390/jcm10122694/s1, Table S1: The sensitivities of the assays, Table S2: Variables used for multivariate logistic regression analysis, Table S3: Multivariate logistic regression model of early recurrence atrial fibrillation created in the group of men.

## References

[1] Calkins H., Hindricks G., Cappato R., Kim Y.H., Saad E.B., Aguinaga L., Akar J.G., Badhwar J., Brugada J., Camm J. (2018). 2017 HRS/EHRA/ECAS/APHRS/SOLAECE expert consensus statement on catheter and surgical ablation of atrial fibrillation. Europace.

[2] Budzianowski J., Hiczkiewicz J., Burchardt P., Pieszko K., Rzeźniczak J., Budzianowski P., Korybalska K. (2019). Predictors of atrial fibrillation early recurrence following cryoballoon ablation of pulmonary veins using statistical assessment and machine learning algorithms. Heart Vessel..

[3] Korybalska K., Luczak J., Swora-Cwynar E., Kanikowska A., Czepulis N., Kanikowska D., Skalisz H., Bręborowicz A., Grzymisławski M., Witowski J. (2017). Weight loss-dependent and-independent effects of moderate calorie restriction on endothelial cell markers in obesity. J. Physiol. Pharmacol..

[4] Lang R.M., Badano L.P., Mor-Avi V., Afilalo J., Armstrong A., Ernande L., Flachskampf F.A., Foster E., Goldstein S.A., Kuznetsova T. (2015). Recommendations for cardiac chamber quantification by echocardiography in adults: An update from the American society of echocardiography and the European association of cardiovascular imaging. Eur. Heart J. Cardiovasc. Imaging.

[5] Coglianese E.E., Larson M., Vasan R.S., Ho J.E., Ghorbani A., McCabe E.L., Cheng S., Fradley M.G., Kretschman D., Gao W. (2012). Distribution and Clinical Correlates of the Interleukin Receptor Family Member Soluble ST2 in the Framingham Heart Study. Clin. Chem..

[6] Lew J., Sanghavi M., Ayers C.R., McGuire D.K., Omland T., Atzler D., Gore M.O., Neeland I., Berry J.D., Khera A. (2017). Sex-Based Differences in Cardiometabolic Biomarkers. Circulation.

[7] Okar S., Kaypakli O., Şahin D.Y., Koç M. (2018). Fibrosis Marker Soluble ST2 Predicts Atrial Fibrillation Recurrence after Cryoballoon Catheter Ablation of Nonvalvular Paroxysmal Atrial Fibrillation. Korean Circ. J..

[8] Chamberlain A.M., Agarwal S.K., Folsom A.R., Duval S., Soliman E.Z., Ambrose M., Eberly L., Alonso A. (2011). Smoking and incidence of atrial fibrillation: Results from the Atherosclerosis Risk in Communities (ARIC) Study. Heart Rhythm..

[9] Cheng W.-H., Lo L.-W., Lin Y.-J., Chang S.-L., Hu Y.-F., Hung Y., Chung F.-P., Chang T.-Y., Huang T.-C., Yamada S. (2018). Cigarette smoking causes a worse long-term outcome in persistent atrial fibrillation following catheter ablation. J. Cardiovasc. Electrophysiol..

[10] Godfraind T. (2017). Discovery and Development of Calcium Channel Blockers. Front. Pharmacol..

[11] Providência R., Adragão P., de Asmundis C., Chun J., Chierchia G., Defaye P., Anselme F., Creta A., Lambiase P.D., Schmidt B. (2019). Impact of Body Mass Index on the Outcomes of Catheter Ablation of Atrial Fibrillation: A European Observational Multicenter Study. J. Am. Heart Assoc..

[12] Ricciardi D., Arena G., Verlato R., Iacopino S., Pieragnoli P., Molon G., Manfrin M., Allocca G., Cattafi G., Sirico G. (2019). Sex effect on efficacy of pulmonary vein cryoablation in patients with atrial fibrillation: Data from the multicenter real-world 1STOP project. J. Interv. Card. Electrophysiol..

[13] Andrade J.G., Khairy P., Macle L., Packer D.L., Lehmann J.W., Holcomb R.G., Ruskin J.N., Dubuc M. (2014). Incidence and significance of early recurrences of atrial fibrillation after cryoballoon ablation: Insights from the multicenter Sustained Treatment of Paroxysmal Atrial Fibrillation (STOP AF) Trial. Circ. Arrhythmia Electrophysiol..

[14] Fukui A., Takahashi N., Nakada C., Masaki T., Kume O., Shinohara T., Teshima Y., Hara M., Saikawa T. (2013). Role of Leptin Signaling in the Pathogenesis of Angiotensin II—Mediated Atrial Fibrosis and Fibrillation. Circ. Arrhythmia Electrophysiol..

[15] Demerath E., Towne B., Blangero J., Siervogel R.M. (2001). The relationship of soluble ICAM-1, VCAM-1, P-selectin and E-selectin to cardiovascular disease risk factors in healthy men and women. Ann. Hum. Biol..

[16] Mertens I., Van der Planken M., Corthouts B., Van Gaal L.F. (2006). Is visceral adipose tissue a determinant of von Willebrand factor in overweight and obese premenopausal women?. Metabolism.

[17] Glover B.M., Hong K.L., Dagres N., Arbelo E., Laroche C., Riahi S., Bertini M., Mikhaylov E., Galvin J., Kiliszek M. (2018). Impact of body mass index on the outcome of catheter ablation of atrial fibrillation. Heart.

[18] Miyazaki S., Taniguchi H., Nakamura H., Takagi T., Iwasawa J., Hachiya H., Iesaka Y. (2015). Clinical Significance of Early Recurrence After Pulmonary Vein Antrum Isolation in Paroxysmal Atrial Fibrillation—Insight into the Mechanism. Circ. J..

[19] Gunawardene M.A., Hoffmann B.A., Schaeffer B., Chung D.-U., Moser J., Akbulak R.O., Jularic M., Eickholt C., Nuehrich J., Meyer C. (2016). Influence of energy source on early atrial fibrillation recurrences: A comparison of cryoballoon vs. radiofrequency current energy ablation with the endpoint of unexcitability in pulmonary vein isolation. Europace.

[20] Kızılırmak F., Gokdeniz T., Gunes H.M., Demir G.G., Cakal B., Guler G.B., Guler E., Olgun F.E., Kilicaslan F. (2017). Myocardial injury biomarkers after radiofrequency catheter and cryoballoon ablation for atrial fibrillation and their impact on recurrence. Kardiol. Pol..

[21] Wójcik M., Janin S., Kuniss M., Berkowitsch A., Erkapic D., Zaltsberg S., Madlener K., Wysokiński A., Hamm C.W., Pitschner H.F. (2011). Limitations of Biomarkers Serum Levels During Pulmonary Vein Isolation. Rev. Esp. Cardiol..

[22] Andrade J.G., Champagne J., Dubuc M., Deyell M.W., Verma A., Macle L., Leon-Sit P., Novak P., Badra-Verdu M., Sapp J. (2019). Cryoballoon or radiofrequency ablation for atrial fibrillation assessed by continuous monitoring: A randomized clinical trial. Circulation.

[23] Lim H.S., Schultz C., Dang J., Alasady M., Lau D.H., Brooks A.G., Wong C.X., Roberts-Thomson K.C., Young G.D., Worthley M.I. (2014). Time Course of Inflammation, Myocardial Injury, and Prothrombotic Response After Radiofrequency Catheter Ablation for Atrial Fibrillation. Circ. Arrhythmia Electrophysiol..

