# Supplementary material for: Predictors of Early-Recurrence Atrial Fibrillation after Catheter Ablation in Women and Men with Abnormal Body Weight
Source: J Clin Med. 2021 Jun 18;10(12):2694. doi: 10.3390/jcm10122694 (PMC8235463; doi:10.3390/jcm10122694)
Supplement: Supplementary file 1 [file jcm-10-02694-s001.zip › jcm-1251742-supplementary.pdf]

Supplementary Materials

Table S1. The sensitivities of the assays

Parameter	Sensitivity
hsIL-6 pg/ml	0.04 pg/ml
Pentraxin (PTX) ng/ml	0.06 ng/ml
vWF ng/ml	0.10 ng/ml
sICAM-1 ng/ml	0.02 ng/ml
sVCAM-1 ng/ml	0.01 ng/ml
Thrombomodulin (TM) ng/ml	0.03 ng/ml
t-PA ng/ml	0.10 ng/ml
PAI-1 ng/ml	0.14 ng/ml
ST2 ng/ml	0.04 ng/ml
Leptin ng/ml	0.03 ng/ml
Adiponectin μg/ml	0.93 μg/ml
D-dimer	0.5 μg/ml
Hs-TnT	14 pg/ml
CRP	0.5 mg/dl

vWF, von Willebrandt factor; *TM*, thrombomodulin; *s-ICAM*, intercellular adhesion molecule*; v-ICAM*, vascular cell adhesion molecule*; t-PA,* tissue plazminogen activator; *PAI,* plasminogen activator inhibitor-1; hs-TnT, high-sensitive cardiac troponin T; *CRP,* C-reactive protein

Table S2. Variables used for multivariate logistic regression analysis.

No	VARIABLES
1	Coronary artery disease
2	Hypertension
3	Thyroid diseases
4	Dyslipidemia
5	Heart Failure
6	Anti-arrhytmic drugs
7	Oral anticoagulants
8	Beta-blokers
9	Statins
10	ACEI
11	Sartans
12	Calcium channel blockers
13	Diuretics
14	Smoking
15	HAS-BLED score
16	CHA2DS2-VASC score
17	EHRA score
18	Sex
19	Radiofrequency ablation
20	Cryoballoon ablation
21	Age
22	Weight
23	BMI
24	WC
25	Adiponektin
26	Leptin
27	Leptin to adiponectin ratio
28	Na
29	K
30	Glukose
31	Cholesterol
32	HDL
33	LDL
34	Triglyceride
35	TSH
36	Urea
37	Creatinine
38	GFR
39	AlAT
40	AspAT
41	Procedure time (min)
42	Fluoroscopic time (min)
43	Number of application (min)
44	Application time (min)
45	LA volume
46	LAVI
47	EF

Table S2. *Cont.*

**48**	SBP after ablation<br>SBP delta
49	DBP after ablation<br>DBP delta
50	hs IL-6 after ablation<br>hs IL-6 delta
51	CRP after ablation<br>CRP delta
52	Pentraxin after ablation<br>Pentraxin delta
53	WBC after ablation<br>WBC delta
54	Hs-Troponin T after ablation<br>Hs-Troponin T delta
55	CPK after ablation<br>CPK delta
56	CK-MB after ablation<br>CK-MB delta
57	vWF after ablation<br>vWF delta
58	TM after ablation<br>TM delta
59	sVCAM-1 after ablation<br>sVCAM-1 delta
60	sICAM-1 after ablation<br>sICAM-1 delta
61	t-PA – after ablation<br>t-PA-delta
62	PAI-1 after ablation<br>PAI-1 delta
63	PLT after ablation<br>PLT delta

Table S2. *Cont.*

64	Fibrinogen after<br>Fibrinogen delta
65	D-dimer after ablation<br>D-dimer delta
66	ST2 after ablation<br>ST2 delta

ACE-I, angiotensin converting enzyme inhibitor; *CHA_2DS_2-VASc*, Congestive heart failure, Hypertension, Age ≥ 75 (doubled), Diabetes, Stroke (doubled), Vascular disease, Age 65–74, Sex (female); *HAS-BLED,* Hypertension, Abnormal renal/liver function, Stroke, Bleeding history or predisposition, Labile INR, Elderly (>65 years), Drugs/alcohol concomitantly; *EHRA,* European Heart Rhythm Association; *BMI*, body mass index; *WC* – waist circumference; *GFR,*glomerular filtration rate; *LAVI,* left atrial volume index; *EF*, ejection fraction; *INR*, international normalized ratio; *SBP,*Systolic blood pressure; *DBP,* Diastolic blood pressure; *CRP,* C-reactive protein; *PLT,* platelets;*WBC,* white blood count; hs-TnT, high-sensitive cardiac troponin T; *vWF,* von Willebrandt factor; *TM*, thrombomodulin; *s-ICAM*, intercellular adhesion molecule; *v-ICAM*, vascular cell adhesion molecule; *t-PA,* tissue plazminogen activator; *PAI,* plasminogen activator inhibitor-1

Table S3. Multivariate logistic regression model of early recurrence atrial fibrillation created in the group of men.

Variables	P value	OR	95 % CI
ST2 delta	0,011	1,68	1,115 – 2,536
CCB	<0.05	0,042	0,002 - 1,071
smoking	<0.05	39,25	1,050 – 1467,8

OR: odds ratio; *CI:* confidence interval; *CCB:* calcium channel blockers; *CI:* confidence intervals
